# Hierarchy of non-glucose sugars in Escherichia coli

**DOI:** 10.1186/s12918-014-0133-z

**Published:** 2014-12-24

**Authors:** Guy Aidelberg, Benjamin D Towbin, Daphna Rothschild, Erez Dekel, Anat Bren, Uri Alon

**Affiliations:** Department of Molecular Cell Biology, Weizmann Institute of Science, Rehovot, Israel

**Keywords:** *E. coli*, Carbon catabolic repression, CCR, Diauxic shift, Non-PTS sugars, Cellular decision making, cAMP, CRP, CAP

## Abstract

**Background:**

Understanding how cells make decisions, and why they make the decisions they make, is of fundamental interest in systems biology. To address this, we study the decisions made by *E. coli* on which genes to express when presented with two different sugars. It is well-known that glucose, *E. coli’s* preferred carbon source, represses the uptake of other sugars by means of global and gene-specific mechanisms. However, less is known about the utilization of glucose-free sugar mixtures which are found in the natural environment of *E. coli* and in biotechnology.

**Results:**

Here, we combine experiment and theory to map the choices of *E. coli* among 6 different non-glucose carbon sources. We used robotic assays and fluorescence reporter strains to make precise measurements of promoter activity and growth rate in all pairs of these sugars. We find that the sugars can be ranked in a hierarchy: in a mixture of a higher and a lower sugar, the lower sugar system shows reduced promoter activity. The hierarchy corresponds to the growth rate supported by each sugar- the faster the growth rate, the higher the sugar on the hierarchy. The hierarchy is ‘soft’ in the sense that the lower sugar promoters are not completely repressed. Measurement of the activity of the master regulator CRP-cAMP shows that the hierarchy can be quantitatively explained based on differential activation of the promoters by CRP-cAMP. Comparing sugar system activation as a function of time in sugar pair mixtures at sub-saturating concentrations, we find cases of sequential activation, and also cases of simultaneous expression of both systems. Such simultaneous expression is not predicted by simple models of growth rate optimization, which predict only sequential activation. We extend these models by suggesting multi-objective optimization for both growing rapidly now and preparing the cell for future growth on the poorer sugar.

**Conclusion:**

We find a defined hierarchy of sugar utilization, which can be quantitatively explained by differential activation by the master regulator cAMP-CRP. The present approach can be used to understand cell decisions when presented with mixtures of conditions.

**Electronic supplementary material:**

The online version of this article (doi:10.1186/s12918-014-0133-z) contains supplementary material, which is available to authorized users.

## Background

Cells need to make decisions when faced with multiple options. It is of general interest to understand principles which guide cell decision making, and to understand whether the decisions made are optimal in some sense [1-3]. To address this, we focus on the choices that *E. coli* makes when presented with more than one carbon source.

When multiple carbon sources are available bacteria can either co-metabolize them or preferentially use one of the carbon sources before the others. The best known example of preferential carbon utilization comes from the work of Monod on the glucose-lactose diauxic shift in *E. coli* [4]. Bacteria first utilized only glucose, and when glucose ran out, switched to lactose.

Subsequent studies revealed that glucose is the preferred carbon source for many organisms [5]. The presence of glucose often prevents the use of secondary carbon sources. This phenomena is termed glucose repression or more generally carbon catabolic repression (CCR) [6]. CCR is a central regulatory mechanism that affects 5-10% of all genes in many bacterial species ([5,7-10] for reviews).

CCR is believed to be important in natural environments to allow the bacteria to grow rapidly on its preferred sugar. On the other hand, in industrial processes such as biofuel production from sugar mixtures (such as agricultural byproducts), CCR is one of the barriers for increased yield of fermentation processes [11].

The molecular mechanism underlying CCR in *E. coli* has been worked out for the class of sugars transported by the phosphotransferase system (PTS) sugars, including glucose and mannose. The transport pathway leads to reduced levels of a key signaling molecule, cyclic AMP (cAMP). cAMP, in turn, binds the global regulator CRP which activates most carbon utilization promoters. Thus, PTS sugars lower CRP activity, and lead to inactivation of alternative carbon systems. In addition, transport through PTS transporters leads to direct inhibition of several sugar pumps ([5,7-10], for reviews). Recently, post transcriptional control by small regulatory RNA (sRNA) has also been discovered to play a role in CCR [12,13].

The contribution of each of these mechanisms to CCR is probably different for different carbon sources and is debated even for the best studied CCR example of the glucose-lactose diauxie shift [14,15]. The level of cAMP in the cell is also determined by the metabolic and energetic state of the cell [16,17]. Central carbon metabolites (α-ketoacids) can negatively affect cAMP levels when nitrogen availability is low, thus forming an integral feedback loop that can control carbon uptake to match cell needs between anabolism and catabolism [10,18,19].

In contrast to the extensive knowledge on the preferential utilization of glucose [7], much less is known about the utilization of glucose-free sugar mixtures, especially on mixtures of non-PTS sugars. These non-PTS sugars are often found in the environmental niches of *E. coli*. Sugars found in the intestinal habitat of *E coli* have been characterized, and cases of sequential and simultaneous utilization of these sugars have been reported in complex mixtures of these sugars [20,21]. This hints at the existence of a secondary hierarchy of sugar utilization.

The mechanism for a non-PTS sugar hierarchy was directly addressed in *E. coli* for the mixture of arabinose and xylose. These sugars, together with glucose, are the main components of lignocelluloses, which is a substrate for bacterial biofuel production. Desai et al. [22] showed that arabinose consumption precedes xylose consumption, and that xylose utilization genes are partially inhibited in the presence of arabinose and xylose. They further proposed that the xylose utilization promoters are directly repressed by the arabinose specific transcription factor AraC [22]. There is need for further systematic study of sugar secondary hierarchies and their mechanism, in order to better understand the decisions that *E. coli* makes in complex nutrient conditions.

Here, we combine experiments and theory to map the sugar utilization hierarchy of *E. coli* for 6 different non-PTS carbon sources. We find a defined hierarchy in the activation of sugar systems, where the promoter of the less dominant sugar system has reduced activity. The ranking of the sugars in the hierarchy is the same as the ranking of the growth rate supported by the sugars as sole carbon sources. The hierarchy can be quantitatively explained by differential CRP-cAMP activation of the promoters. Both sequential and simultaneous expression of sugar systems is found when one of the sugars is at low concentration, suggesting a multi-objective optimization strategy for decision making in sugar mixtures.

## Results

### Sugar utilization promoter activities were measured in all pairs of six non-PTS sugars

To study growth and promoter activity in sugar mixtures we used a robotic assay with fluorescence reporter strains. The reporter strains were taken from a comprehensive *E. coli* reporter library [23], in which a full length promoter region controls fast-folding GFP (gfpmut2) on a low copy plasmid. We studied six non-PTS sugars with well characterized catabolic systems: α-lactose, L-arabinose, D-xylose, D-sorbitol, D-ribose and L-rhamnose [24]. Each system was represented by a promoter for one of its utilization operons: *lacZYA*, *araBAD*, *xylAB*, *srlAEBD*, *rbsDACBKR* and *rhaBAD* respectively. All promoters showed strong expression during exponential growth on their cognate sugars (signal/background fluorescence ratios of 3–20, see Methods).

Cells were grown in 96-well plates in an automated shaking incubator. GFP fluorescence and cell density were measured every 6–12 minutes over 20 hours of growth. Each measurement was done in at least two replicate wells, and repeated at least on two different days from freshly grown cells. Promoter activity was calculated as the rate of fluorescence change per OD unit as described [25]. Mean day-day relative errors of growth rate and promoter activity were 5% and 11% respectively (see Methods).

### Promoter activity shows a hierarchy of dominance among sugars

We first measured growth and promoter activity on each sugar alone at saturating concentrations (0.2%). The sugars provided different maximal growth rates, ranging between 0.53 ± 0.01 h^−1^on lactose to 0.29 ± 0.01 h^−1^ on ribose at mid exponential phase (Additional file 1: Figure S1).

We then studied mixtures of all of the 15 pairs of these six sugars at saturating concentrations (0.2%). We measured the promoter activity of each sugar system at mid-exponential phase, by averaging the promoter activity over a window of two generations centered at the point of maximal growth rate.

**Figure 1:**
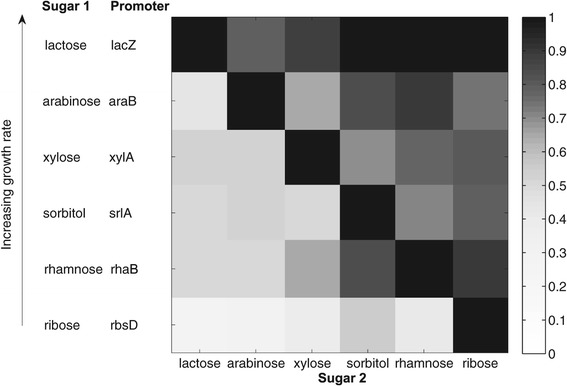
**A hierarchy of sugar gene expression matches the hierarchy in growth rate.** Promoter activity for six different sugar utilization operons at mid exponential growth, in the presence of the cognate sugar alone or paired with each of the 5 other sugars. All sugars are at saturating concentrations (0.2%). Rows represent the promoter activity from the indicated reporter grown in the presence of its cognate sugar. Rows are ordered according to growth rate, with a sugar supporting higher growth as sole carbon source rate located in an upper row. Columns represent the second sugar in the mixture. The diagonal represents the presence of only the cognate sugar (0.2%); promoter activity values in each row were normalized to this value.

We find that the expression of sugar system promoters shows a hierarchy. In the presence of two sugars, the promoter of the sugar supporting lower growth rate is suppressed (Figure 1). Repression is not complete, promoter activity ranges between 0.1-0.5 of the maximal activity of that system growing on its cognate sugar alone. The dominant sugar system shows nearly full expression in the presence of the less dominant sugar.

For example, the highest sugar in the hierarchy among the six sugars in this study, lactose, reduces the expression of all other sugar promoters (left column Figure 1). No other sugar, when mixed with lactose, causes a significant reduction in *lacZYA* promoter activity (top row Figure 1). The lowest sugar in the hierarchy, ribose, barely reduces the activity of any other sugar promoter when mixed with their cognate sugar (right column Figure 1). In contrast, the ribose system promoter, shows very low activity (10-20% of its maximum) when ribose is mixed with any other sugar in the set (bottom row Figure 1).

The ranking of the hierarchy based on promoter activity is the same as the ranking according to the growth rate supported by the sugars as sole carbon sources: lactose > arabinose > xylose > sorbitol > rhamnose > ribose. In Figure 1, the sugars are arranged according to the growth rate order; the upper-triangular form of the expression matrix, with high values mainly above the diagonal, is a graphic representation of the hierarchy. We obtained equivalent results when we normalized gene expression by growth rate or by the activity of a synthetic σ^70^ reporter that reflects global transcriptional activity (Additional file 1: Figures S2, S3). Thus, the observed sugar hierarchy is not caused by global effects on gene regulation due to changes in growth rate [26-28].

It should be noted that the growth rate in the mixtures is equal to the growth rate of the higher sugar alone to within experimental accuracy, except for mixtures containing ribose which grow 3-15% faster than with either sugar alone (Additional file 1: Figure S1, see below).

### Sugar systems show very little cross activation

**Figure 2:**
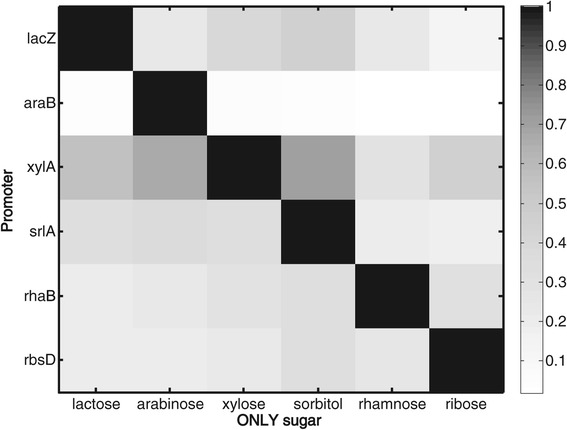
**Sugar system promoters show very little cross regulation.** Promoter activity for six different sugar utilization promoters in the presence of only one sugar, at saturating concentration (0.2%) at mid-exponential phase. Rows represent the reporter genes and columns represent the sugar in the medium. Promoter activity of each reporter gene was normalized to the activity in its cognate sugar.

We also tested the extent of cross-activation of a system by non-cognate sugars. In this case we measured promoter activity of the same 6 promoters above, in each of the studied sugars alone at saturating concentration (0.2%). We find high expression of a promoter only when grown on its cognate sugar – the diagonal in Figure 2. Promoters had low activity in the presence of non-cognate sugars as the sole carbon source (median of 20% of their activity in their cognate sugar). This indicates little cross-activation between different sugar systems. Thus, promoters without their cognate sugars cannot be appreciably activated by non-cognate sugars. However, as shown in Figure 1, a system turned ON by its cognate sugar can be substantially down-regulated in the presence of non-cognate sugars in the mixture. This deactivation occurs if the non-cognate sugar is higher in the hierarchy (supports faster growth).

### Sugar hierarchy can be quantitatively explained by differential activation by cAMP-CRP

**Figure 3:**
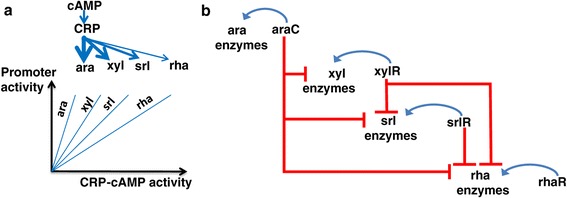
**Two possible regulatory mechanisms that can implement a hierarchal decision in sugar utilization. a)** Hierarchy can be obtained if CRP shows differential regulation for the different sugar systems so that the induction curves of each system as a function of CRP-cAMP activity are separated. **b)** Hierarchy can also be obtained by cross regulation so that systems lower in the hierarchy are directly repressed, for example by the sugar-specific transcription factors of the better sugar systems.

Two non-mutually-exclusive hypotheses for the mechanism for the observed hierarchy are (i) that a single global regulator, such as CRP-cAMP, controls sugar choices by differential regulatory input functions to each system (Figure 3a) and (ii) That a network of cross regulation exists where a low-ranking sugar system is repressed by the regulator of the higher ranking sugar (Figure 3b).

To address possible mechanisms for the hierarchy, we measured the activity of a CRP reporter strain. CRP-cAMP is a transcriptional activator of all of the sugar promoters in this study [24,29]. We used a reporter strain for CRP-cAMP activity, which carries a reporter plasmid with a CRP-cAMP responsive promoter. The promoter is based on the *lacZ* promoter with the LacI binding sites reshuffled, described in [30]. We find that CRP-cAMP reporter activity at mid-exponential phase on different sugars increases linearly with cell generation time (data not shown), in accordance with previous studies [17,18].

**Figure 4:**
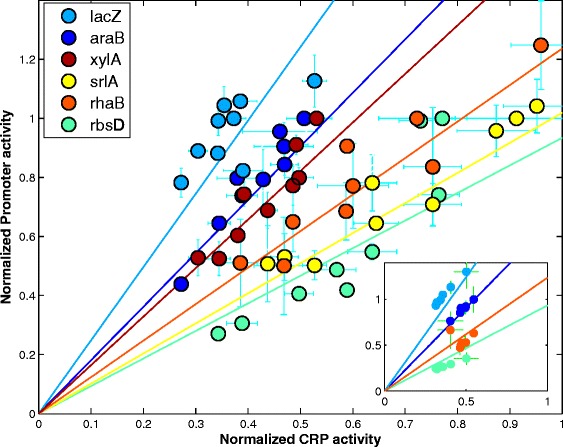
**Sugar system promoters show a linear increase with CRP-reporter activity but with different slopes that match the hierarchy.** Promoter activity at mid exponential phase of each sugar system promoter in the presence of its cognate sugar and one of the five other sugars, normalized to when only its cognate sugar is present, as a function of the promoter activity of a CRP reporter normalized to its highest value. Each color represents a different sugar system promoter (*lacZ* light blue, *araB* blue, *xylA* brown, *rhaB* orange, *srlA* yellow, *rbsD* green). Inset: promoter activity at mid-exponential phase in a two sugar mixture in the presence of external cAMP at 0,0.15,0.3,0.6,1.25,2.5,5 mM. The promoters are lacZ and rbsD measured with external cAMP in lactose + ribose; araB and rhaB measured with external cAMP in arabinose + rhamnose.

We next plotted the sugar system promoter activities measured in all sugar pairs as a function of the CRP-cAMP reporter activity in the same conditions (Figure 4). We find that promoter activity for each sugar system shows an approximately linear increase with CRP reporter activity. The lines have different slopes, such that the lower the sugar on the hierarchy, the lower the slope. Thus, lower sugar systems need more CRP activity to reach high promoter activity. To activate the lowest sugar system, ribose, to its maximal level requires about 3 times more CRP reporter activity than the highest system, lactose. This suggests a way in which differential activation by CRP can implement the hierarchical dominance between sugars (Figure 3a).

We also measured promoter activity in the presence of externally added cAMP, for two of the mixtures (arabinose + rhamnose and lactose + ribose). External cAMP is able to enter the cells and activate CRP [31]. We find that external cAMP causes promoter activity to increase in a linear fashion with CRP-reporter activity (Figure 4 inset), following approximately the same linear relationship obtained by mixing different sugars.

**Figure 5:**
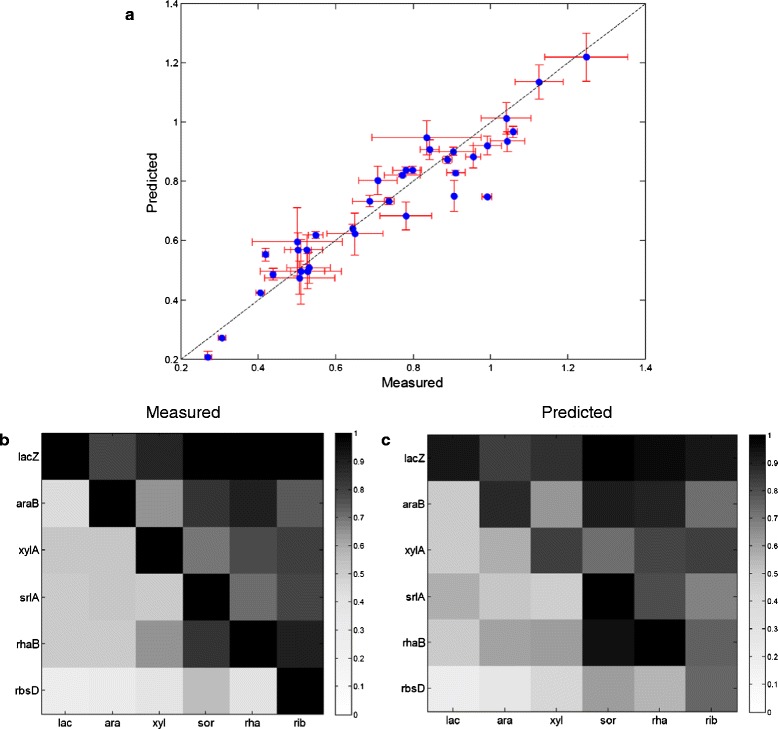
**Differential activation by cAMP-CRP can quantitatively explain the sugar utilization hierarchy. a)** Plotted is the predicted normalized promoter activity versus the measured one. The two agree well with a correlation coefficient R^2^ = 0.95, p < 10^-10^. The error bars are standard errors of 4 biological repeats (x-coordinate error bar) and 95% confidence interval of fits (y-coordinate error bar) **b)** measured promoter activity (same as figure 1) **c)** predicted promoter activity from linear fits to CRP input functions of each promoter (data of Fig 4, predicted points removed from data used for fit).

We asked whether the CRP-cAMP dependence of the sugar promoters is sufficient to explain the observed hierarchy in quantitative detail. We used linear regression lines (methods) to calculate the promoter activity in sugar pairs, using the measured CRP-cAMP reporter activity. We removed the data points we wish to predict from the regression analysis to avoid concerns of circularity. We then predicted promoter activity based on measured CRP reporter activity in each sugar mixture. We find excellent quantitative agreement between the predicted and observed hierarchy (R^2^ = 0.95) (Figure 5a). This concordance is also seen in Figure 5b and c, which compare the measured and predicted promoter activity in a matrix format. This analysis suggests that differential activation by CRP-cAMP can quantitatively explain much of the observed sugar hierarchy.

We also tested the effect of mutating the CRP site in a promoter. We made four point mutations in the CRP binding site in the *rhaB* promoter on the reporter plasmid (Additional file 1). The four mutations brought the CRP site close (to within two mutations) to its consensus sequence [24], which we assumed would enhance the ability of CRP to activate expression. This mutant promoter showed CRP dependent activity with a 30% higher slope than the un-mutated promoter. This effectively moves the *rhaB* promoter from a low to a middle place in the hierarchy (Additional file 1: Figure S4), close to xylose. This mutant experiment supports a causative role for CRP-cAMP in determining the location of promoters in the hierarchy.

As a further control, we used a sugar known to have specific regulation in a sugar mixture, maltose. Maltose system expression is enhanced in the presence of lactose [32,33]. In *E. coli*’s natural environment, the human gut, the appearance of maltose often follows lactose. It has therefore been suggested that co-expression of the two systems prepares *E. coli* for the future maltose presence when feeding on lactose [34]. We find that maltose, when mixed with the six sugars in this study, fits into the hierarchy picture, and lies at a central position in the hierarchy. Two exceptions are lactose and sorbitol (Additional file 1: Figure S5). With lactose, *malP* promoter activity is ~30% higher than predicted based on cAMP-CRP activity in line with published data from [32,33]. In the presence of sorbitol, the expression of the mal system is ~85% lower than expected- it is almost fully repressed. This may indicate a yet unknown regulatory link between the sorbitol and maltose systems.

Finally, we note that the sugar levels used in this experiment are saturating (0.2%). Control experiments show that growth rate and promoter activities are not affected by reducing sugar concentrations by tenfold (0.02%) (Additional file 1: Figure S7), indicating that the cognate regulators are saturated with inducer. This suggests that the observed regulatory variation is not due to variation in inducer levels.

### Both sequential and simultaneous expression of sugar systems is observed

**Figure 6:**
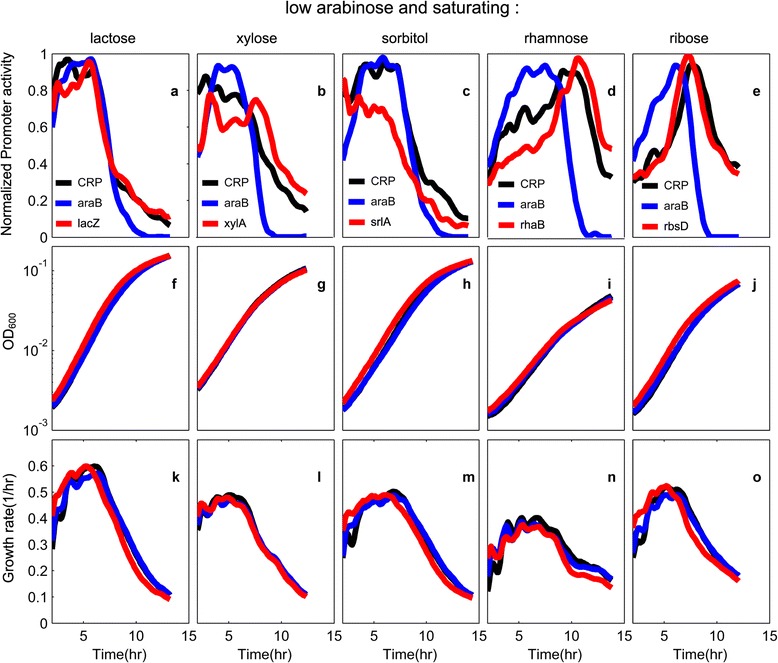
**Different sugar promoters can be either simultaneously or sequentially expressed in a sugar mixture. a-e)** Promoter activity of CRP reporter (black), *araB* (blue), and a second sugar system promoter (red) in a mixture of sub-saturating arabinose (0.005%) and saturating second sugar (0.2%). The second sugars and promoters are **a)** *lacZ* and lactose, **b)** *xylA* and xylose, **c)** *srlA* and sorbitol, **d)** *rhaB* and rhamnose, **e)** *rbsD* and ribose. Note that a, b and c show simultaneous expression of the two promoters, whereas d and e show sequential expression. Also shown are optical density OD_600_ **(f-j)**, and growth rate defined as dlog(OD)/dt panels **(k-o)** for the corresponding growth conditions. Drop in growth rate at late times is entry to stationary phase. Colors represent the strains as in fig a-e. Promoter activity data is normalized to its maximal value, mean day-day relative errors of growth rate and promoter activity were 8% and 9% respectively.

So far, we analyzed sugar gene expression at saturating concentrations of the sugars (0.2%) and at mid-exponential phase. We next asked about the dynamics of sugar system activation, by following the promoter activity as a function of time. We tested five mixtures: arabinose at low concentration (0.005%) mixed with saturating lactose, xylose, sorbitol, rhamnose and ribose (Figure 6). Two of the mixtures- arabinose with rhamnose or ribose (Figure 6 d and e) - showed sequential activation of the sugar promoters. The second promoter gets fully activated at about the same time that the first sugar promoter (*araB*) becomes deactivated. The rise in the second promoter parallels the rise in the activity of the CRP reporter (black curves d and e).

In contrast, the other three sugar mixtures (arabinose with lactose, xylose or sorbitol Figure 6 a, b and c) show simultaneous expression of the two sugar systems (Figure 6). This simultaneous expression parallels a rather steady CRP activity profile (Figure 6 a-c black curves). Thus, sequential activation occurred with sugars lower on the hierarchy, and simultaneous activation with sugars higher on the hierarchy.

Since our assay measures population averages, we cannot distinguish between simultaneous expression of two sugar promoters in every cell and the occurrence of subpopulations with distinct gene expression. To distinguish between these two scenarios we measured fluorescence of the same reporter strains at the level of individual cells, by flow cytometry (Additional file 1). We find that the cell-cell distributions of GFP fluorescence are unimodal (Additional file 1: Figure S6). Thus, in cases of co-expression of two sugar utilization systems, all individual cells seem to express both systems and the population average is a good estimate for the single cell mean.

### Simultaneous expression cannot be explained by simple optimality models, and suggests multi-objective optimality

We finally consider these results in the context of mathematical models to understand the decisions made by *E .coli* on which sugar to utilize, based on analyzing the optimal decisions under given constraints. This approach was presented in the 1980s’ by Kompala, Ramakrishna, Tsao and co-workers [35-38], to model microbial responses to a mixed sugar environment. They proposed a view in which cells are considered to be optimal control systems which maximize a certain goal, namely biomass production. This model as well as more recent studies [39] predict ”bang-bang” control, where only one or the other substrate is utilized. These models were later extended [40,41], to account for new observations of simultaneous utilization where the growth rate is higher on the two substrates together than the maximal growth rate on either substrate alone. For a detailed comparison of these models see [42].

**Figure 7:**
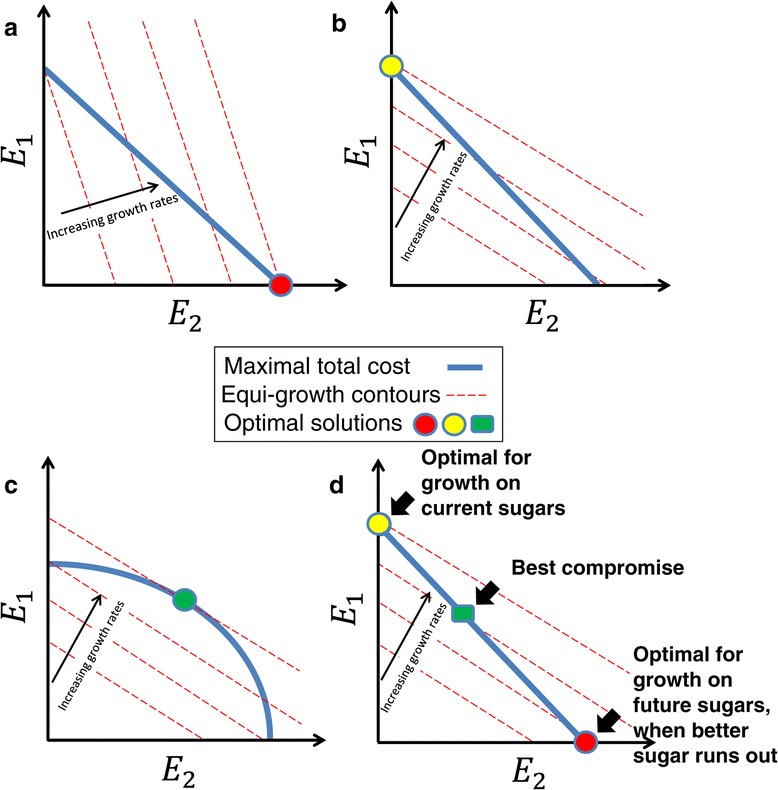
**Simple linear programming optimality models predict that utilizing a single sugar is optimal; more complex models can allow co-utilization of both sugars. a)** Simplified linear programming model: The growth rate increases with the expression of the two sugar systems, E1 and E2 – dashed contours. Given a cost constraint of total proteins (blue line), expressing only one of the two sugar systems maximizes the growth rate (red dot). As the concentration of that sugar decreases, growth rate contours shift their slope, until a point in time is reached when **b)** the optimal solution jumps to expressing the other sugar system exclusively (yellow dot). **c)** If the constraint (blue line) is convex, the constraint curve bulges outwards and co-expression of the two sugar systems can be optimal (green dot). This predicts that growth rate in co-expression exceeds the maximal growth rate expressing each system alone. **d)** Co-expression can also be optimal if tasks other than immediate rapid growth affect fitness, for example future growth on the poorer sugar. The green box symbolizes a potential best compromise solution.

In the models, one compares the benefit brought to the cell in terms of growth rate, to the cost or burden of producing and maintaining the sugar systems [3,43-48]. In the simplest case, the cost of producing *E*_*i*_ proteins of sugar system *i* is described by a reduction in growth rate *cost = a*_*i*_*E*_*i*_, where *a*_*i*_ is the cost per protein in terms of growth rate reduction. The benefit can be modeled as an increase in growth rate due to the action of the sugar enzymes on their substrates: μ = ∑ *S*_*i*_*E*_*i*_*b*_*i*_ where *S*_*i*_ is the sugar concentration (or an increasing function of sugar level such as a Michaelis-Menten relation), and *b*_*i*_ is the contribution to growth rate per protein made by sugar system *i*. In the case of two available sugars, we seek the best expression profile - the values of *E*_*1*_ and *E*_*2*_ that maximize growth rate, given a certain maximal total cost (number of proteins). The optimal solution is all-or-none: either make only E_1_ or only E_2_. The reason is shown graphically in Figure 7a and b, in which the cost constraint is a straight line *a*_*1*_*E*_*1*_ *+ a*_*2*_*E*_*2*_ *= const*, and growth contours are also straight lines of equal μ = ∑ *S*_*i*_*E*_*i*_*b*_*i*_. This situation means that the maximum growth can only be obtained at one of the two corners of the resulting triangular region, at which one system is expressed and the other is fully repressed.

This simple analysis suggests that *E. coli* should choose to consume only one sugar - the sugar that supports higher growth - and express only its system. As this sugar is utilized and its concentration decreases, there comes a critical point when the cell should switch to making only the other sugar system. Graphically, this happens when the contours of growth rate shift their slope, such that beyond a critical slope the solution ‘jumps’ to the other corner of the triangle (Figure 7b). The cell switches from making only E_1_ to making only E_2_. Thus, only sequential activation is predicted by this model, as is indeed observed in the diauxic shift from glucose to lactose, or from arabinose to rhamnose or ribose (Figure 6 d and e).

The observation of simultaneous expression of two sugar systems under some conditions (e.g. Figure 6 a-c) in this and previous studies [20,49], cannot be explained by the model in its simplest form. There are at least two ways in which the model can be modified to allow for co-expression of two systems. One is a constraint line that bulges outward (Figure 7c), as would happen if the cost of two different proteins was smaller than the cost of twice the same protein. This predicts, that co-expression allows a higher growth rate than in the presence of only a single sugar [41]. The present data, however, suggests that in cases of co-expression, growth rate is not measurably higher than in the saturating sugar alone (Additional file: Figure S1, with the exception of one sugar, ribose, discussed below). This generally discounts the nonlinear-constraint explanation of simultaneous expression. The same considerations discount models in which the equi-growth curves (benefit functions) are nonlinear.

A second possibility is that growth rate is not the only component of fitness relevant to evolution of sugar choice. In other words, that sugar system activation decisions are a multi-objective optimization problem [50-53]. One may consider, for example, that *E. coli* devotes part of its resources to prepare for future situations [33,54], *e.g.* when the better sugar runs out. For example, in the presence of lactose and arabinose, it might be useful to co-express the arabinose system in order to shorten the lag phase that is expected to occur after the cells consume the lactose [55], enter stationary phase, and attempt to restart growth on newly arrived arabinose [56,57].

An additional possible multi-objective task is the secondary use of the sugar molecule as a structural material, beyond its use as a carbon and energy source. This may occur in the case of D-ribose, which can be used directly to make nucleotides as a substrate of the enzymes ribose mutarotase and ribokinase [58]. Utilization of external ribose requires its transport and phosphorylation, performed by genes on the ribose operon. In this case, co-expression of the ribose operon together with genes for sugars higher on the hierarchy can result from the need to balance sugar catabolism with direct production of nucleotides, and thus can increase the growth rate, making the equi-growth contours concave.

A final possibility is that the choices of *E. coli* are not always optimal [1].

## Discussion

We find that non-PTS sugars can be ranked in a hierarchy in which the higher sugar partially inhibits the expression of the lower sugar systems. The hierarchy corresponds to the relative growth rate supported by each sugar- the faster the growth rate, the higher the sugar on the hierarchy. The precise promoter activity level in each combination can be quantitatively explained by differential activation of each promoter by the master carbon regulator CRP-cAMP. Mutations in the CRP site of a sugar system promoter can reprogram its position in the hierarchy. In terms of dynamics, we find cases of both sequential activation of the sugar systems, and simultaneous activation in which both systems are expressed at the same time [20,21,40,41,49]. Sequential activation is known to be optimal for maximizing growth, whereas simultaneous activation suggests a multi-objective optimality framework for understanding *E. coli*’s decision making in sugar mixtures.

A hierarchy of N = 6 sugars means that the result of mixing all N(N-1)/2 = 15 pairs of sugars can be explained by 6 numbers - the relative growth rate ranking of the sugar in the hierarchy. This in turn seems to stem from the slope of the CRP-cAMP input function for each promoter. Such a hierarchical decision could in principle be achieved by an alternative design: an intricate network of cross regulation, where a low-ranking sugar system is repressed by the regulator of the higher ranking sugar; this requires numerous repressive binding sites, especially at the lower ranking promoters (Figure 3b).

The global-regulator design suggested here may allow rapid evolutionary tuning of the hierarchy if repositioning of the sugars in the hierarchy is needed. This tuning can occur for example by mutations in the CRP binding regions of a promoter [59-61], changing its input function slope, as demonstrated here for the *rhaB* promoter. Similar alterations might improve the efficiency of biotechnological systems that require growth on sugar mixtures. Inefficient growth has been recently addressed by growing multiple strains together, each of which can only utilize a single carbon source [62].

It would be fascinating to extend this study to other microorganisms, to see if a similar sugar utilization hierarchy exists, and if it is encoded in an analogous way. A differently ordered hierarchy might indicate differences in the availability and usefulness of the specific sugars in the evolutionary environment of different species. It would also be interesting to test whether a hierarchy is found also for the utilization of other essential elements such as nitrogen, sulfur and phosphorus: there are multiple sources which can be utilized and a way of choosing between them may be programmed into the cell. If a hierarchy exists, it would be interesting to see if it is encoded by a single master regulator. The present approach can be used to investigate such questions.

## Conclusions

We mapped the sugar utilization hierarchy of *E. coli* for 6 different non-PTS carbon sources. We find a defined hierarchy in the activation of sugar systems. The promoter of the less dominant sugar system are less active in the presence of the more dominant sugar. The ranking of the hierarchy is the same as the ranking of the growth rate supported by the sugars as sole carbon sources. The hierarchy can be quantitatively explained by differential CRP-cAMP activation of the promoters. Both sequential and simultaneous expression of sugar systems is found, suggesting a multi-objective optimization strategy for decision making in sugar mixtures.

## Methods

### Strains and Growth conditions

All strains in this study are from the library previously described in [23] except for the synthetic cAMP-CRP and σ^70^ activity reporters described in [30], and a *rhaB* reporter with mutated CRP site constructed here (Additional file 1). Briefly, each strain in the library has the native promoter region of a specific operon driving the expression of a rapidly folding green fluorescent protein gene (GFP) optimized for bacteria (gfpmut2) with a strong ribosome binding site, on a low copy plasmid (pSC101 origin) which also harbors a kanamycin resistance gene. All strains in this study were derivatives of wild type *E. coli* K12 strain MG1655.

Growth medium was M9 defined minimal medium (42 mM Na_2_HPO_4_, 22 mM KH_2_PO_4_, 8.5 mM NaCl, 18.7 mM NH_4_Cl, 2 mM MgSO_4_, 0.1 mM CaCl), supplemented with 50 μg/ml kanamycin, as well as the indicated concentration of the different carbon sources and cAMP.

### Robotic assays

Indicated reporter strains were grown overnight in M9 minimal medium containing 0.2% glucose, 0.05% casamino acids, and 50 μg/ml of kanamycin with shaking at 37°C. Using a robotic liquid handler (FreedomEvo, Tecan), 96-well plates were prepared with 150 μl of M9 minimal medium with sugars as indicated. The wells were inoculated with bacteria at a 1:500 dilution from the overnight culture. Wells were then covered with 100 μl of mineral oil (Sigma) to prevent evaporation, a step that we previously found not to significantly affect aeration or growth [63,64], and transferred into an automated incubator. Cells were grown in the incubator with shaking (6 Hz) at 37°C for about 20 hr, the incubator contained up to 9 different plates. Every ~12 min (or ~6 min when 4 plates were used instead of nine) the plate was transferred by a robotic arm into a multi-well fluorimeter (Infinite F200, Tecan) that reads the OD (600 nm) and GFP (535 nm)

### Promoter Activity and growth rate calculation

Data was obtained from plate reader software (Evoware, Tecan) and processed using custom Matlab software as described [25]. Background fluorescence was subtracted from GFP measurements using a reporter strain bearing promoterless vector pUA66 for each well. Promoter activity was then calculated using the temporal derivative of GFP divided by the OD_600_. Growth rate was calculated as the temporal derivative of the natural logarithm of the OD curves, α = *d*ln(OD)/*dt*. Mid log phase was defined by a region of 2 generations centered around the point of maximal growth rate. The present assay based on GFP from plasmid-borne promoters has a lower dynamic range than LacZ-based assays and other methods, as discussed in [65]. This is due to the fact that we cannot resolve the low expression state, and thus we cannot achieve a ratio of 300–1000 between the high and low ends of the expression range of some promoters. However, the results in this study are at the high end of the expression range of the promoters, because we use saturating sugars for Figures 1, 2 and 4. At the high end, there should be no compression or nonlinear effect, making the present assay suitable for the questions asked here.

### CRP Model fitting

To predict promoter activity of a specific promoter in a specific combination of sugars, a line was fit to the measured normalized promoter activity of the specific promoter as a function of the normalized promoter activity of the CRP reporter using least squares regression, without taking into consideration the data of the point we wanted to predict.

## Additional file

Additional file 1: Figure S1. Growth rates in pairs of sugars. **Figure S2.** A hierarchy in gene expression exists and it matches the hierarchy in growth rate- data of Figure 2 in main text normalized for growth rate. **Figure S3.** A hierarchy in gene expression exists and it matches the hierarchy in growth rate-data of Figure 1 of main text, normalized to growth rate. **Figure S4.** Mutations that improve the CRP site in the rhaB promoter reporter plasmid lead to a rise of the CRP-dependent activation slope. **Figure S5.** Maltose system lies in the middle of the hierarchy, but has two outlier sugar mixtures. **Figure S6.** Single cell GFP measurements are unimodal. **Figure S7.** Mid-exponential growth rate and promoter activity are robust to changes in cognate sugar concentration in the range 0.2%-0.02%.
