# Supplementary material for: Hierarchy of non-glucose sugars in Escherichia coli
Source: BMC Syst Biol. 2014 Dec 24;8:133. doi: 10.1186/s12918-014-0133-z (PMC4304618; doi:10.1186/s12918-014-0133-z)
Supplement: Additional file 1: Figure S1. — Growth rates in pairs of sugars. Figure S2. A hierarchy in gene expression exists and it matches the hierarchy in growth rate- data of Figure 2 in main text normalized for growth rate. Figure S3. A hierarchy in gene expression exists and it matches the hierarchy in growth rate-data of Figure 1 of main text, normalized to growth rate. Figure S4. Mutations that improve the CRP site in the rhaB promoter reporter plasmid lead to a rise of the CRP-dependent activation slope. Figure S5. Maltose system lies in the middle of the hierarchy, but has two outlier sugar mixtures. Figure S6. Single cell GFP measurements are unimodal. Figure S7. Mid-exponential growth rate and promoter activity are robust to changes in cognate sugar concentration in the range 0.2%-0.02%. [file 12918_2014_133_MOESM1_ESM.docx]

**Additional File 1:**

**Hierarchy of non-glucose sugars in Escherichia coli**

Guy Aidelberg, Benjamin D. Towbin, Daphna Rothschild, Erez Dekel, Anat Bren and Uri Alon

Supplementary information


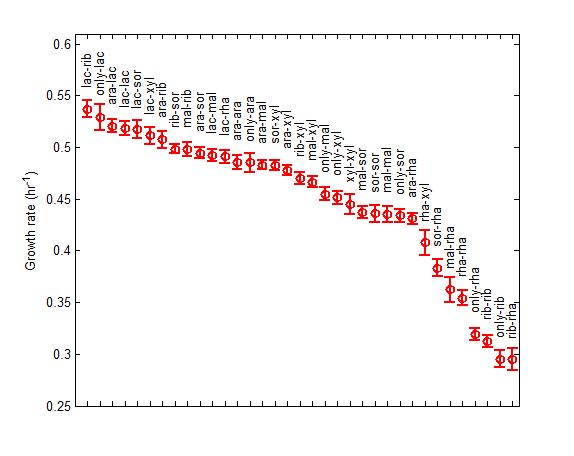


**Supplementary figure 1: Growth rates in pairs of sugars. a)** Measured growth rates of different sugar pairs, during mid-exponential growth phase at saturating concentration of 0.2% of the indicated sugar; the same sugar twice indicates a concentration of 0.4%.

**Supplementary Figure 2: A hierarchy in gene expression exists and it matches the hierarchy in growth rate- data of Fig 2 in main text normalized for growth rate.** The figure shows promoter activity for 6 different sugar utilization genes in the presence of the cognate sugar alone or paired with 5 other sugars. Each row represents the promoter activity from the indicated reporter gene grown in the presence of the cognate sugar. Rows are ordered according to growth rate, with a sugar supporting higher growth rate located in an upper row. Each column represents the second sugar in the medium. The diagonal represents the presence of only the cognate sugar; promoter activity values in each row were normalized to this value, and then to the activity of a reporter measuring the activity of σ70;

**Supplementary Figure 3:** **A hierarchy in gene expression exists and it matches the hierarchy in growth rate-data of Fig 1 of main text, normalized to growth rate.** The figure shows promoter activity for six different sugar utilization genes in the presence of the cognate sugar alone or paired with 5 other sugars. Each row represents the promoter activity from the indicated reporter gene grown in the presence of the cognate sugar. Rows are ordered according to growth rate, with a sugar supporting higher growth rate located in an upper row. Each column represents the second sugar in the medium. The diagonal represents the presence of only the cognate sugar; promoter activity values in each row were normalized to this value and then also by the specific growth rate in the combination;

**
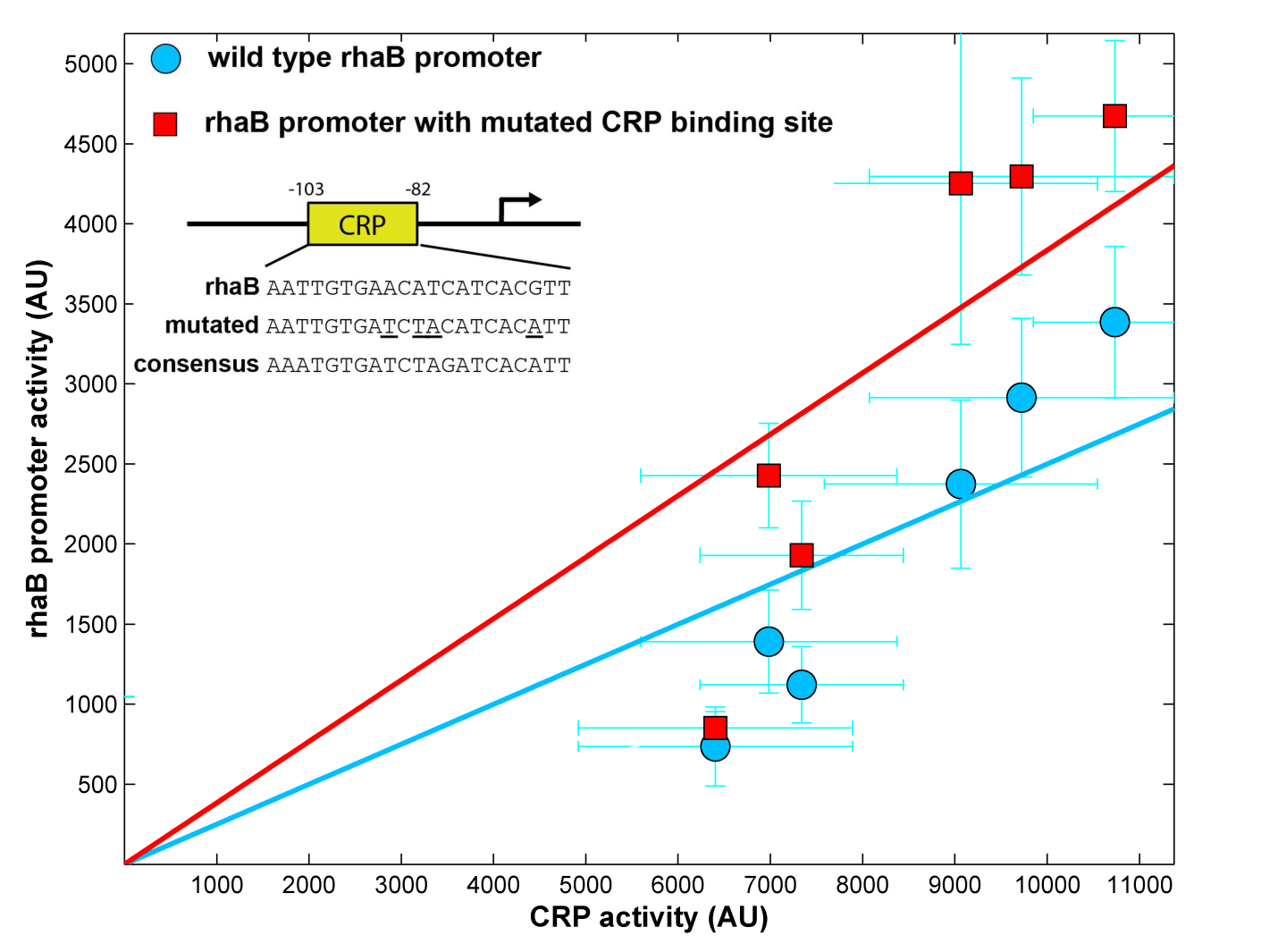
**

**Supplementary Figure 4: Mutations that improve the CRP site in the rhaB promoter reporter plasmid lead to a rise of the CRP-dependent activation slope.**

Mutated CRP binding site in the rhaB promoter reporter plasmid was constructed by site directed mutagenesis using the QuikChange® II Site-Directed Mutagenesis kit (Stratagene, Catalog #200524), with primers TCAGCAAATTGTGATCTACATCACATTCATCTTTCCCTGG and CCAGGGAAAGATGAATGTGATGTAGATCACAATTTGCTGA. The wild type sequence at position -103 is AATTGTGAACATCATCACGTT. We mutated the underlined positions to result in a new site AATTGTGATCTACATCACATT. This brings the site to be within 2 mutations of the consensus CRP site, AAATGTGATCTAGATCACATT, thought to correspond to strongest CRP binding [1]. We measured the wild type and mutant reporter strains in all 6 combinations of rhamnose and a second sugar (including double the rhamnose dose), at 0.2% concentration. Promoter activity at the maximal exponential growth rate was evaluated as a function of CRP reporter activity in the same conditions. Experiment was done in 8 replicate wells, with two day-day repeats. The mutated promoter showed a slope as a function of CRP reporter activity that is about 30% higher than the wild-type reporter (Fig 4). This indicates that the stronger CRP site in the mutant led to a rise in its place on the hierarchy.


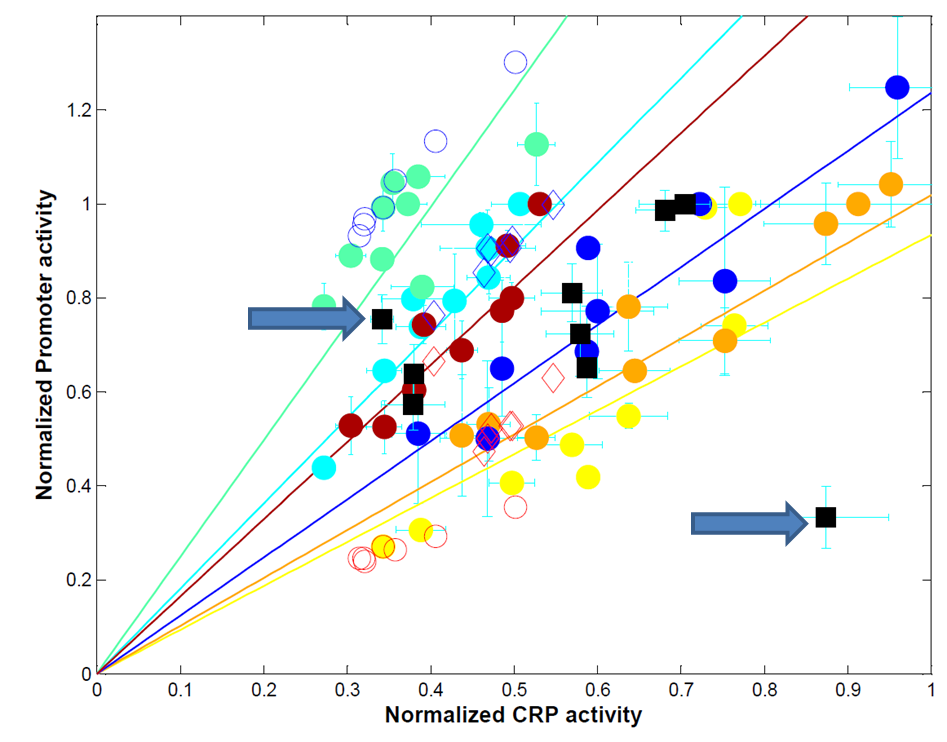


**Supplementary Figure 5: Maltose system lies in the middle of the hierarchy, but has two outlier sugar mixtures.** Black squares: promoter activity at mid exponential phase, versus CRP reporter activity, in mixtures of maltose plus the six other sugars of this study at saturating concentrations (0.2%). Highlighted are mixtures with lactose and sorbitol, which fall away form the best fit line. Also shown are the linear fits to the input functions of the six promoters in the main text (Fig 4). Promoter activity is normalized to its value in the cognate sugar alone; CRP reporter activity is normalized to its maximal value.


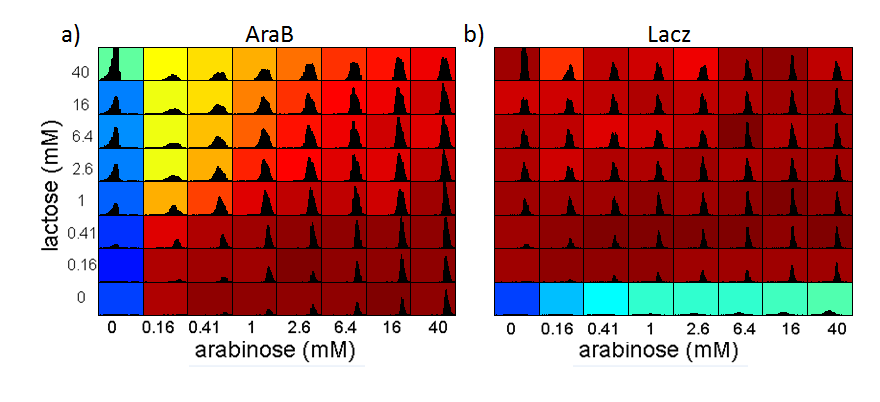


**Supplementary Figure 6: Single cell GFP measurements are unimodal.** a) araB promoter b) lacZ promoter. Histograms of the log GFP level of 5000 to 50000 individual cells, after 8 hours of growth in different concentrations of the two sugars. x and y axes correspond to starting sugar concentration (40mM) diluted in steps of 2.5. Color corresponds to the mean GFP florescence level of the cells in each well.

**Single cell (FACS) Methods**

Samples were prepared in 96 well plates as described in main text, but at 8 different concentrations of the 2 sugars (1:2.5 serial dilutions of arabinose and lactose). Plates were covered with a breathable film/membrane and a lid. Cells were grown in an incubator with shaking (6 Hz) at 37°C for 8 hours and then transferred to the flow cytometer. 20 µl sample at a flow rate of 1 µl/s were analyzed per sample using a LSRII (BD, USA) flow cytometry instrument (settings: FSC 648 mV, SSC 346 mV, GFP 450 mV, threshold collection FSC > 200) at 4 °C. The cells' FSC, SSC and GFP values were collected. The events rate for the bacterial samples was between 1,000-9,500 events/s, and did not come near the upper limit of the instrument (20,000 events/s), to avoid coincidence [2, 3]. A total of 5,000-50,000 events were collected per sample. FSC & SSC were used for gating. In contrast to most flow cytometry experiments that usually use narrow gating to achieve measurements of a homogenized population, a wide gating was used in this study, this large gating allowed measuring the entire population.


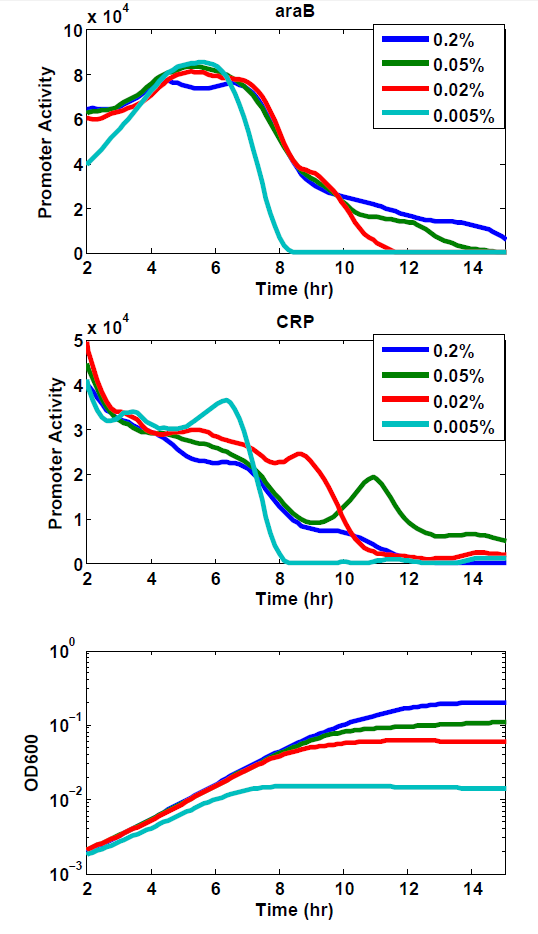


**a)**

**c)**

**b)**

**Supplementary Figure 7: Mid-exponential growth rate and promoter activity are robust to changes in cognate sugar concentration in the range 0.2%-0.02%.** a) araB promoter activity. b) CRP promoter activity. c) And optical density OD_600_. Figure shows promoter activity of the araB and CRP reporter, as well as the OD at 0.2%, 0.05%, 0.02% and 0.005% arabinose as the sole carbon source. Note that at all concentrations the peak of araB promoter activity is about the same. This indicates that at these concentrations the inducer may saturate its corresponding TF at the phase of maximal growth rate.

Bibliography:

1. Keseler IM, Mackie A, Peralta-Gil M, Santos-Zavaleta A, Gama-Castro S, Bonavides-Martínez C, Fulcher C, Huerta AM, Kothari A, Krummenacker M, Latendresse M, Muñiz-Rascado L, Ong Q, Paley S, Schröder I, Shearer AG, Subhraveti P, Travers M, Weerasinghe D, Weiss V, Collado-Vides J, Gunsalus RP, Paulsen I, Karp PD: **EcoCyc: fusing model organism databases with systems biology.** *Nucleic Acids Res* 2013, **41**(Database issue):D605–12.

2. Marie D, Vaulot D, Partensky F: **Application of the novel nucleic acid dyes YOYO-1, YO-PRO-1, and PicoGreen for flow cytometric analysis of marine prokaryotes.** *Appl Environ Microbiol* 1996, **62**:1649–55.

3. Gasol JM, Del Giorgio PA: **Using flow cytometry for counting natural planktonic bacteria and understanding the structure of planktonic bacterial communities**. *Sci Mar* 2008, **64**:197–224.
